# The clinical impact of patients with bloodstream infection with different groups of Viridans group streptococci by using matrix-assisted laser desorption ionization–time of flight mass spectrometry (MALDI-TOF MS)

**DOI:** 10.1097/MD.0000000000013607

**Published:** 2018-12-14

**Authors:** Ting-Yi Su, Ming-Hsun Lee, Ching-Tai Huang, Tsui-Ping Liu, Jang-Jih Lu

**Affiliations:** a Division of Infectious Diseases, Department of Internal Medicine, Chang Gung Memorial Hospital at Linkou, Chang Gung University College of Medicine; b Department of Laboratory Medicine, Chang Gung Memorial Hospital at Linkou; c Department of Medical Biotechnology and Laboratory Science, Chang Gung University, Taoyuan, Taiwan.

**Keywords:** blood stream infection, clinical impact, MALDI-TOF MS, Viridans group streptococci groups

## Abstract

The accuracy of matrix-assisted laser desorption ionization–time of flight mass spectrometry (MALDI-TOF MS) for identifying viridans group streptococcus (VGS) was improving. However, the clinical impact of identifying VGS had not been well recognized. Our study had comprehensively studied the clinical manifestations and outcome of VGS blood stream infection by using MALDI-TOF MS for identification.

This retrospective study enrolled 312 adult patients with a monomicrobial blood culture positive for VGS. Blood culture was examined through MALDI-TOF MS.

The most common VGS species were the *Streptococcus anginosus* group (38.8%) and *Streptococcus mitis* group (22.8%). Most species showed resistance to erythromycin (35.6%), followed by clindamycin (25.3%) and penicillin (12.5%). Skin and soft tissue infection and biliary tract infection were significantly related to *S. anginosus* group bacteremia (*P* = .001 and *P* = .005, respectively). *S. mitis* group bacteremia was related to infective endocarditis and bacteremia with febrile neutropenia (*P* = .005 and *P* < .001, respectively). Infective endocarditis was also more likely associated with *S. sanguinis* group bacteremia (*P* = .009). *S. anginosus* group had less resistance rate to ampicillin, erythromycin, clindamycin, and ceftriaxone (*P* = .019, <.001, .001, and .046, respectively). A more staying in intensive care unit, underlying solid organ malignancy, and a shorter treatment duration were independent risk factors for 30-day mortality. This study comprehensively evaluated different VGS group and their clinical manifestations, infection sources, concomitant diseases, treatments, and outcomes. Categorizing VGS into different groups by MALDI-TOF MS could help clinical physicians well understand their clinical presentations.

## Abstract 2

Supplemental Digital Content is available in the text

## 1 Introduction

Viridans group streptococci (VGS) include different species of organisms that can be both commensal flora and pathogens in humans.^[1]^ VGS are considered normal flora of the oropharyngeal, urogenital, and gastrointestinal microbiota.^[2]^ Identification of VGS to the species level can be difficult, and previous phenotypic identification was not always accurate with controversial taxonomy, diverse clinical manifestations, and “poorly classified” diseases. In 1997, only 15 different streptococcal species were included in VGS.^[3]^ With the development of techniques such as matrix-assisted laser desorption ionization–time of flight mass spectrometry (MALDI-TOF MS),^[4–6]^ and 16S rRNA, sod A gene, or gyrB gene sequencing,^[6–8]^ the accuracy of the identification of VGS to the species level has increased significantly. The accuracy of MALDI-TOFMS for identifying VGS to the group level was generally reliable.^[6,9]^ Currently, VGS are classified into 6 major groups: *Streptococcus mutans*, *Streptococcus salivarius*, *Streptococcus anginosus*, *Streptococcus mitis*, *Streptococcus sanguinis*, and *Streptococcus bovis* groups.^[10]^

VGS have been reported to have relationships with some specific diseases in children, including a significant relationship with infective endocarditis^[11–13]^ and bacteremia in pediatric patients with cancer, particularly in febrile and neutropenic patients.^[14–16]^ Some species, such as *S. mitis* or *S. sanguinis*, have been reported to be more likely to cause infective endocardititis,^[17,18]^ and several studies have demonstrated a relationship between *S. mitis* and bacteremia in patients with febrile neutropenia.^[14,19]^ However, the clinical significance of different VGS group has not been comprehensively studied, especially in adult patients. Thus, to gain an insight into the association of different VGS group with their clinical presentations, diseases, risk factors, and outcomes, we performed a comprehensive review of VGS bloodstream infections in adult patients in a tertiary care medical center between 2014 and 2015.

## 2 Methods

### 2.1 Setting

This retrospective study was conducted at Chang Gung Memorial Hospital (CGMH), Linkou, Northern Taiwan, which is a 3715-bed university-affiliated tertiary-care medical center with 308 intensive care unit (ICU) beds. All clinical specimens were processed using computer-assisted microbiology laboratory databases at a central microbiology laboratory. This study was approved by the Institutional Review Board of CGMH (201600969B0).

### 2.2 Study design and patients

**Figure 1:**
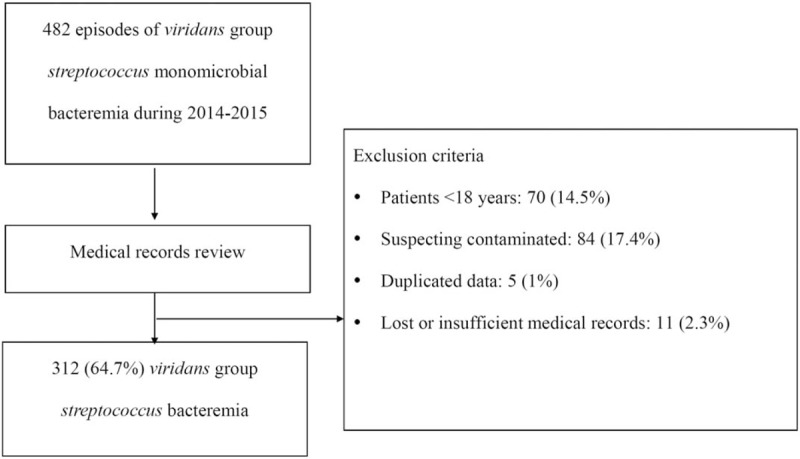
Flow chart of the exclusion of patients with *viridans* group *streptococcus* bacteremia.

This retrospective study reviewed 482 patients admitted to CGMH from January 2014 to December 2015 with a monomicrobial blood culture positive for VGS. Additional inclusion criteria were as follows: being aged ≥18 years, a clinical syndrome suggestive of systemic infection, with clear medical records, and being unduplicated cases. Finally, 312 (64.7%) patients were enrolled (Fig. 1). The patients with VGS bacteremia without a clinical syndrome suggestive of systemic infection were suspected contamination and excluded.

### 2.3 Microbiology

Blood cultures were processed in the clinical microbiology laboratory by using an automated blood culture system (BACTEC 9240 system; Becton Dickinson Diagnostic Instrument Systems, Sparks, MD). Positive blood culture was identified using the MALDI-TOF MS instrument (Bruker Daltonics, Bremen, Germany). Measurements were performed with the Bruker Biotyper MALDI-TOF MS system using FlexControl version 3.4 software with Compass Flex Series version 1.4 software and a 60-Hz nitrogen laser (337 nm wave length). Spectra ranging from 2000 to 20,000 m/z were analyzed using the MALDI Biotyper system's automation control and the current Bruker Biotyper V.3.1 software and library [database (DB)5989 with 5989 entries]. The identification criteria used in our analysis, as outlined by the manufacturer, were as follows: A score of ≥2 indicated identification to the species level, a score between 1.7 and 1.9 indicated identification to the genus level, and a score of ≤1.7 was interpreted as unreliable identification. VGS were classified into 6 main groups: *S. anginosus* group, comprising *S. anginosus*, *S. constellatus*, and *S. intermedius*; *S. mitis* group, comprising *S. mitis, S. oralis*, and *S. infantis*; *S. bovis* group, comprising *S. gallolyticus* and *S. lutetiensis; S. sanguinis* group, comprising *S. sanguinus*, *S. parasangunis*, and *S. gordonii*; *S. salivarius* group, comprising *S. salivarius*; and *S. mutans* group, comprising *S. mutans.*^[2]^ Although *S. pneumoniae* and *S. pseudomonae* also belonged to *S. mitis* group by the DNA association,^[20]^ we excluded them due to the fact that their clinical manifestations are different from the other VGS. *S. pneumoniae* and *S. pseudomonae* were excluded by the results of MALDI-TOF MS and Optochin test. As the previous study did not completely show reliable results of *S. mitis* group identification by MALDI-TOF Biotyper system, we also arranged 16S rRNA sequencing by randomized selected *S. mitis* group.^[6]^ The 16S rRNA gene was amplified for all the isolates using the universal primers with 8FPL (5’-AGTTTGATCCTGGCTCAG-3’) and 1492R (5’-TACGGYTACCTTGTTACGACTT-3’). Species identification was performed by comparing the obtained sequences against those in the Basic Local Alignment Search Tool of GenBank database (http://www.ncbi.nlm.nih.gov/BLAST/). A sequence similarity of 99% was applied as species identification “cut-off” value for the 16S rRNA gene region. Antimicrobial susceptibility testing was performed according to the Clinical and Laboratory Standards Institute interpretive criteria for the disk diffusion method.^[21]^ Antibiotic disks (BD Microbiology Systems, Cockeysville, MD) for VGS included ampicillin, clindamycin, ceftriaxone, erythromycin, penicillin, vancomycin, and teicoplanin.

### 2.4 Data collection and definition

Demographic data, such as age, sex, concomitant diseases, and clinical characteristics, of patients with VGS bacteremia were retrieved by reviewing inpatient medical records. Concomitant diseases included severe renal impairment (defined as chronic kidney disease stages 4 and 5 and requiring renal replacement therapy), diabetes mellitus, cerebrovascular accident, liver cirrhosis, chronic pulmonary disease, and malignancy. Central venous catheter (CVC) placement, ventilator use, ICU stay, immunosuppressant use, and the time interval between hospitalization and occurrence of VGS bacteremia were recorded. Disease severity scores were calculated using the Pittsburgh bacteremia score on the day of VGS bacteremia occurrence.^[22]^ Pittsburgh bacteremia score was calculated as follows: ear temperature: 2 points for a temperature of ≦35°C or ≥40°C, 1 point for a temperature of 35.1°C to 36.0°C or 39.0°C to 39.9°C, and 0 point for a temperature of 36.1°C to 38.9°C; hypotension: 2 points for an acute hypotensive event with decreases in systolic and diastolic blood pressure of >30 and >20 mm Hg, respectively, use of intravenous vasopressor agents, or systolic blood pressure <90 mm Hg; receipt of mechanical ventilation: 2 points; cardiac arrest: 4 points; and mental status: alert, 0 point; disoriented, 1 point; stupor, 2 points; and comatose, 4 points. Severe sepsis was defined as sepsis plus evidence of organ dysfunction, including one of the following criteria: arterial hypoxemia [arterial oxygen tension (PaO_2_]/fraction of inspiration O_2_; FiO2 < 300]; acute oliguria (urine output < 0.5 mL/kg per hour for at least 2 h despite adequate fluid resuscitation); increase in creatinine > 0.5 mg/dL; coagulation abnormalities [international normalized ratio (INR) > 1.5, activated partial thromboplastin time (aPTT) > 60 seconds, and platelets < 100,000/μL]; hepatic dysfunction (elevated bilirubin); paralytic ileus; and decreased capillary refill or skin mottling. Septic shock was defined as sepsis with hypotension refractory to fluid resuscitation. Neutropenia was defined as an absolute neutrophil count of <0.5 × 10^9^/L.

Bacteremia sources determined from medical records, imaging studies, surgical findings, and microbiological evidence were categorized into lower respiratory infection, urinary tract infection, infective endocarditis, bacteremia with febrile neutropenia, uncomplicated skin and soft tissue infection and complicated skin and soft tissue infection (cSSTI), central nervous system infection, central catheter-associated bloodstream infection, and intra-abdominal infections (including biliary tract infection). cSSTI is defined as a heterogeneous package of disorders in healthy people with severe infection, patients with major comorbidities and relatively minor infection, patients with extensive cellulitis and systemic symptoms who can be managed with antibiotics alone to patients with necrotizing limb-threatening infection that requires life-saving surgery, and diabetic foot infections.^[23]^ Febrile neutropenia is defined as an oral temperature of >38.5°C or 2 consecutive readings of >38.0°C for 2 hours and an absolute neutrophil count of <0.5 × 10^9^/L (or expected to fall below 0.5 × 10^9^/L). If no source was identified, the infection was categorized as primary bacteremia. In addition, the infection was classified as primary bacteremia if the blood culture was not consistent with any culture from the site of the suspected infectious focus, including sputum, urine, wound, deep tissue, cerebrospinal fluid, central catheter tip, bile, or ascites.

Treatment duration was defined as the period from the day of appropriate antimicrobial agent use to the end of the treatment. We also recorded patients’ treatment with appropriated antibiotics within 3 days or not, and also the time to appropriate antibiotics. Patients requiring surgical intervention or drainage and the duration between bacteremia occurrence and intervention were also recorded. Clinical outcomes were assessed using 30-day crude mortality.

### 2.5 Statistical analyses

All statistical analyses were performed using the Statistical Package for Social Sciences for Windows (version 18.0; SPSS Inc., Chicago, IL). Categorical variables were compared using the χ^2^ test or Fisher exact test, as appropriate; continuous variables were compared using the Mann–Whitney *U* test. Variables with *P* < .05 in the univariate analysis were included in a multiple logistic regression model by using the backward stepwise method for identifying risk factors for 30-day mortality. Adjusted odds ratios (ORs) and 95% confidence intervals (95% CIs) were calculated. All tests were 2-tailed, and *P* < .05 was considered significant.

## 3 Results

### 3.1 Patient enrollment and their clinical characteristics

A total of 482 VGS blood isolates were identified during 2014 to 2015. On the basis of our inclusion criteria, 170 (35.3%) patients were excluded because they were aged <18 years, or because of suspected contamination, duplicate data, or lost or incomplete medical records. Finally, 312 (64.7%) patients with individual unduplicated VGS blood isolates were enrolled (Fig. 1).

The demographic and clinical characteristics of the patients are listed in Table 1. Of the 312 patients, 64.1% were men with a mean age of 62.4 years. The duration between hospital admission and VGS bacteremia occurrence ranged from 0 to 215 days with a mean interval of 4.1 ± 14.9 days. In total, 71 (22.8%), 65 (20.8%), 40 (12.8%), 66 (21.2%), 37 (11.9%), and 23 (7.4%) patients had received a CVC placement, had received immunosuppressants, ever used a ventilator, had an ICU stay, had severe sepsis or septic shock, and had neutropenia, respectively. The mean Pittsburgh bacteremia score was 1.3 ± 2.7.

**Table 1:**
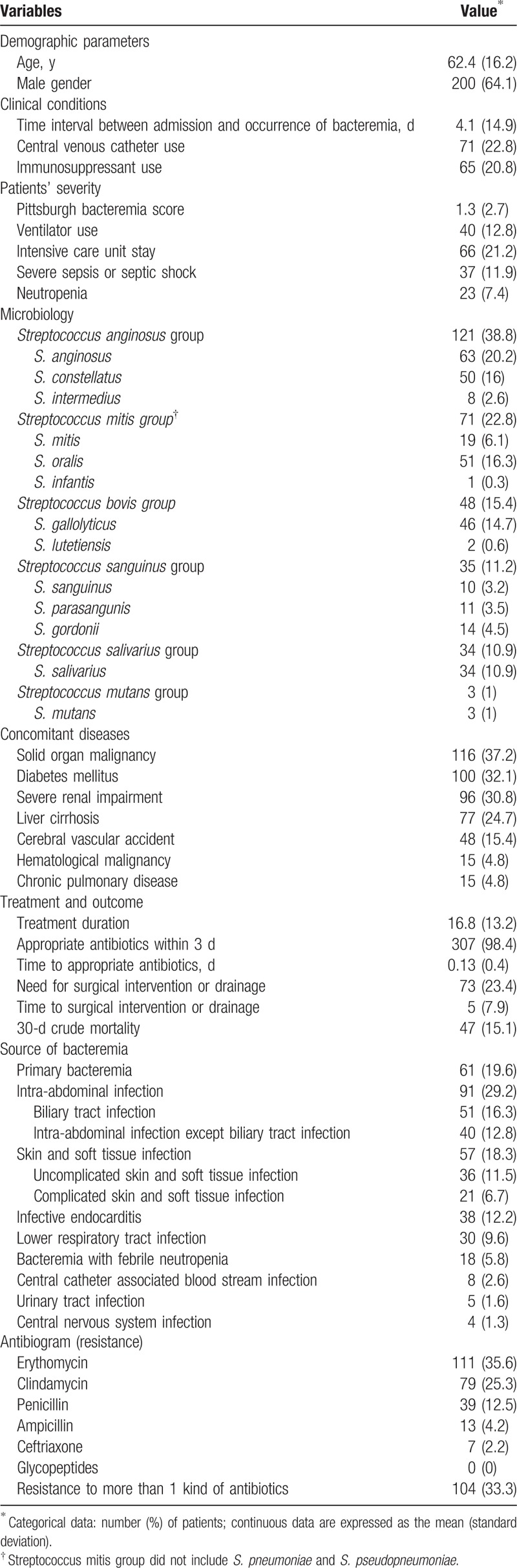
Clinical characteristics of 312 patients with unduplicated viridans group streptococcus monomicrobial bacteremia.

#### 3.1.1 Microbiology, concomitant diseases, treatment, and outcome

This study observed the most common VGS group to be constituted by the *S. anginosus* group with 121 (38.8%) isolates, followed by the *S. mitis* group with 71 (22.8%) isolates. Of the 71 isolates of *S. mitis* group identified by MALDI-TOF MS Biotyper system, we randomized to chose 17 isolates (24%) and confirmed by 16S rRNA sequencing (supplement Table 1). The 2 examination results showed 100% consistency. The most common concomitant disease was solid-organ malignancy (37.2%), followed by diabetes mellitus (32.1%) and chronic kidney disease stage IV and above (30.8%). The treatment duration varied from 0 to 113 days with a mean duration of 16.8 ± 13.2 days; nearly, all 307 (98.4%) patients had received appropriate antibiotics within 3 days. The time to appropriate antibiotics was 0.13 ± 0.4 days. There were 73 (23.4%) patients in need for surgical intervention or drainage, and the duration between bacteremia occurrence and surgical intervention or drainage was 5 ± 7.9 days. Finally, 30-day crude mortality was 15.1%.

### 3.2 Infection sources of different VGS groups

**Figure 2:**
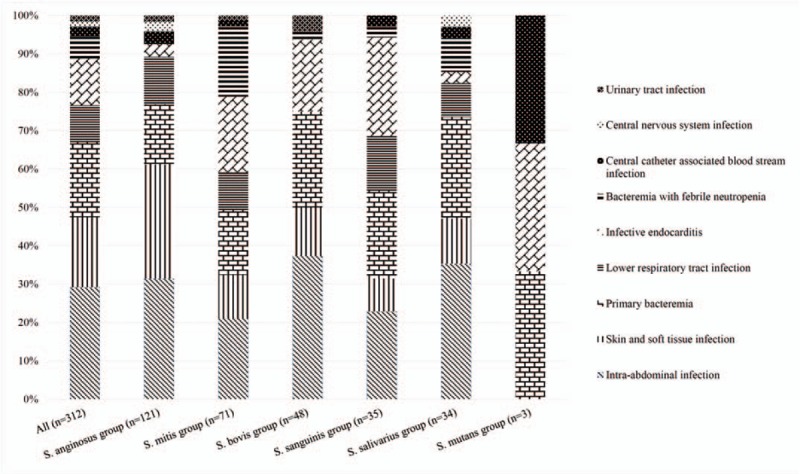
The infection source of different species group of *viridans* group *streptococcus*. ^∗^Mitis group: except *Streptococcus pneumoniae* and *S. pseudopneumoniae*.

Sixty-one (19.6%) patients had primary bacteremia, and the remaining 251 (80.4%) patients had identified sources of bacteremia (Table 1). The most common source of bacteremia was intra-abdominal infection (91/251, 36.34%), followed by skin and soft tissue infection (SSTI; 57/251, 22.7%). Figure 2 depicts the infectious sources of all VGS bacteremia and 6 main VGS groups.

Patients with *S. anginosus* group bacteremia had a higher frequency of SSTI (*P* < .001) and biliary tract infection (*P* = .051) and a lower frequency of infective endocarditis (*P* < .001) and bacteremia with febrile neutropenia (*P* < .001; Table 2). Patients with *S. mitis* group bacteremia had a higher frequency of infective endocarditis (*P* = .027) and bacteremia with febrile neutropenia (*P* < .001) and a lower frequency of biliary tract infection (*P* = .005). Patients with *S. sanguinis* group bacteremia were also more likely to have infective endocarditis (*P* = .009).

**Table 2:**
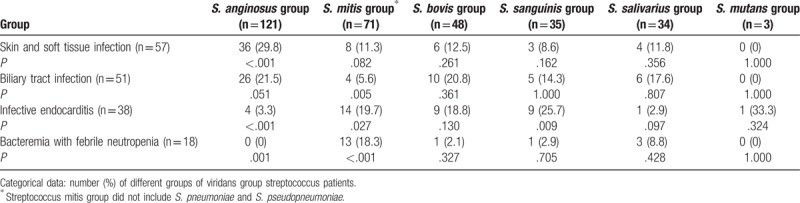
Relationship between different infection sources and different groups of viridans group streptococci.

### 3.3 Antibiotic resistance of different VGS groups

Most patients showed antibiotic resistance to erythromycin (35.6%), followed by clindamycin (25.3%) and penicillin (12.5%; Table 1). One hundred four (33.3%) patients had resistance to more than 1 type of antibiotic. Regarding the antibiotic resistance profiles of different VGS groups, *S. anginosus* group had less resistance to ampicillin, erythromycin, clindamycin, and ceftriaxone (0.8%, 20.7%, 14.8%, and 0%; *P* = .019, <.001, .001, and .046, respectively) (Table 3). *S. bovis* group had higher resistance to erythromycin and clindamycin (54.2% and 50%, *P* = .003 and < .001, respectively) and less resistance to penicillin (0%, *P* = .004). Besides, *S. sanguinis* group had higher resistance to ampicillin (13.7%, *P* = .009) and *S. salivarius* group had higher resistance to penicillin (29.4%, *P* = .002).

**Table 3:**
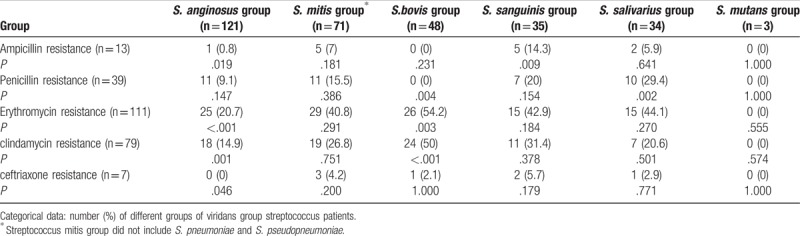
Susceptibility testing of different groups of viridans group streptococci.

### 3.4 Risk factors for 30-day mortality of patients with VGS bacteremia

Significant factors associated with 30-day mortality in the univariate analysis included underlying solid-organ malignancy, severe renal impairment, liver cirrhosis, a longer time interval between admission and bacteremia occurrence, a higher Pittsburgh bacteremia score, more ICU stay, severe sepsis or septic shock, immunosuppressant use and neutropenia, and bacteremia source with lower respiratory tract infection. Infection source with SSTI, longer treatment duration, and more surgical intervention or drainage improved the outcome (Table 4). Different VGS groups and antibiogram and resistance profiles did not reveal a difference in outcomes. The factors associated with 30-day mortality in the univariate analysis were entered into the multivariate analysis, and the results showed that a longer ICU stay (adjusted OR, 6.002; 95% CI, 2.159–16.69; *P* = .001) and underlying solid organ malignancy (adjusted OR, 2.736; 95% CI, 1.09–6.871; *P* = .032) were independent risk factors for 30-day mortality. A longer treatment duration was an independent protective factor for 30-day mortality (adjusted OR, 0.917; 95% CI, 0.866–0.971; *P* = .003; Table 4).

**Table 4:**
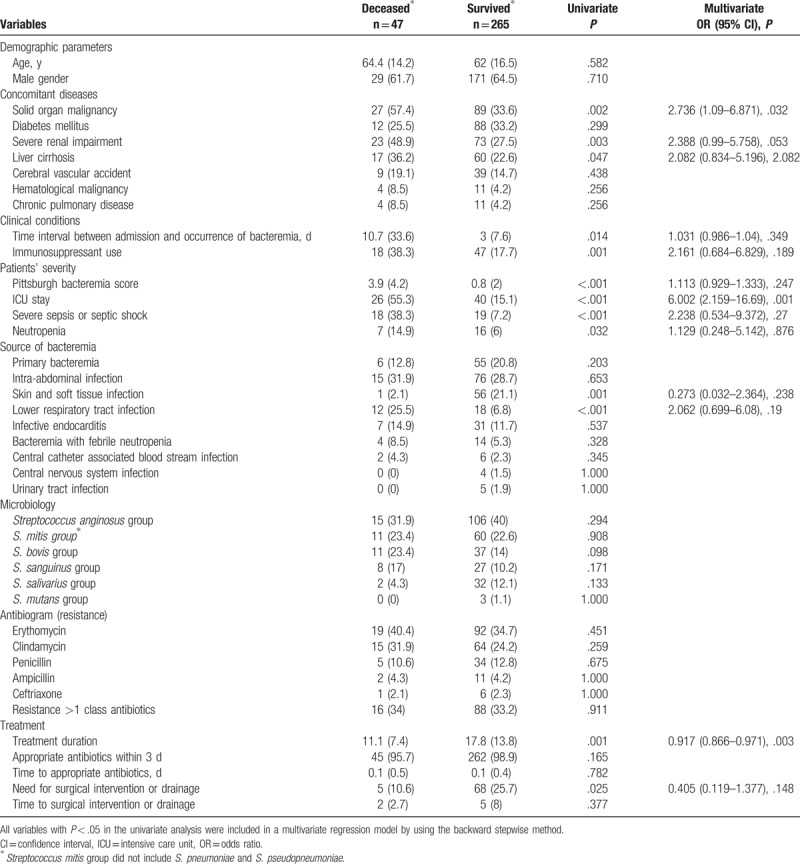
Univariate and multivariate analyses of risk factors for 30-day crude mortality of groups of viridans group streptococcus bacteremia.

## 4 Discussion

Viridans streptococci and the β-hemolytic streptococci constitute a diverse group of organisms with varying environmental niches and pathogenicity. In the previous era, VGS was usually only identified to the genus level due to the limitation of traditional biochemistry tests. Our hospital conducted MALDI-TOF MS for all bacteria identification since June 2013.^[2]^ Although several previous studies had questioned about the ability of MALDI-TOF MS for identifying VGS into species level, especially misidentification *S. mitis* to *S. pneumoniae*, the accuracy for identification to the group level was generally acceptable.^[5,6]^ The updated MALDI Biotyper database showed better performance, provided precise species-level information, and produced no misidentifications of the *S. mitis* species group strains, as *S. pneumoniae* with sensitivity and specificity was closed to 100%.^[24–28]^ Our identification of *S. mitis* group also showed high consistancy results between MALDI-TOF Biotyper and16S rRNA sequencing without misidentification to *S. pneumoniae*. Indeed, using gene sequencing methods might well identify VGS to the species level but is not practical in the real-world settings and limits the ability of clinicians to readily recognize the clinical significance of these VGS. In this study, we did a comprehensive survey of different VGS groups and their clinical manifestations, infection sources, concomitant diseases, treatments, and outcomes in post-MALDI-TOF MS era.

VGS can cause various clinical diseases from infective endocarditis^[11]^ to bacteremia^[29]^ to infection related to malignancy.^[15]^ In the preantibiotic era, VGS accounted for approximately 75% of cases of infective endocarditis. Currently, the relative frequency of VGS in association with infective endocarditis has decreased to as low as 20%.^[30]^ In our study, the *S. mitis* group, *S. bovis* group, and *S. sanguinis* groups were responsible for 36.8%, 23.7%, and 23.7% of infective endocarditis, respectively, with a significantly higher ratio than that of other VGS groups. By contrast, the association of the *S. anginosus* group with infective endocarditis was weak. This finding is supportive of those previous studies.^[17,18,31]^ For accurate diagnosis of infective endocarditis, the identification of VGS is necessary and helpful.

In the setting of a malignancy, bacteremia with VGS is more common in children than in adults, typically developing after chemotherapy or bone marrow transplantation at the time of profound neutropenia. In addition, the relationship between VGS and hematological malignancy or neutropenia was found in adult patients.^[32]^*S. mitis* was the causative organism in most such cases and responsible for severe complications, including shock and acute respiratory failure.^[19,33]^ Although studies specifically evaluating these virulent streptococcal infections in adults are scant, the previously reported incidence ranged from 0% to 26% depending on the population analyzed.^[19]^ In our study, we also found a significant correlation of *S. mitis* group infections and bacteremia with febrile neutropenia.

Earlier observations showed that a striking feature of the *S. anginosus* group was its members’ tendency to cause abscesses.^[34]^ Claridge et al^[34]^ demonstrated that of 60 patients, 22 and 9 had soft tissue infection and intra-abdominal infection, respectively. In our study, the *S. anginosus* group more likely caused pyogenic infection, including intra-abdominal infection (31.4%) and SSTI (29.3%). Although VGS did not constitute the major pathogens of SSTI and intra-abdominal infection, these infections still play a crucial role in *S. anginosus* group bacteremia. *Streptococcal* bone and joint infections are less common than staphylococcal cases. A study reported that the percentages of *S. anginosus* and *S. constellatus* infection were 11% and 10%, respectively, in all 93 patients with *Streptococcal* bone and joint infections.^[35]^ Despite Enterobacteriaceae being the major bacterial cause of intra-abdominal infection, abscess caused by the *S. anginosus* group with a high recurrence rate should still be noted.^[36]^

Although VGS are generally susceptible to most anti-Gram positive agents, resistance occurs against macrolides, tetracycline, and penicillin.^[37]^ The developing penicillin resistance is an emergency in VGS.^[38]^ Alcaide et al^[39]^ indicated that antimicrobial resistance to penicillin differed among these species: *S. mitis*, 41.5%; *S. sanguinis*, 41.7%; *S. salivarius*, 28.1%; and *S. anginosus*, 14% (*P* < .01). Süzük et al^[40]^ reported that the rates of resistance and reduced sensitivity of isolates for penicillin and ampicillin were 61.2% and 55.1%, respectively. The rate of resistance to penicillin and ampicillin was as high as 75% in the *S. sanguinis* group. Chun et al^[41]^ showed that the rates of resistance to penicillin, ampicillin, and ceftriaxone were 40%, 32.8%, and 11.2%, respectively. In our study, the *S. anginosus* group had a low resistance to ampicillin, ceftriaxone, erythromycin, and clindamycin. And *S. bovis* group had the higher resistance to erythromycin (54.2%) and clindamycin (50%), similar with the results of Streit et al.^[42]^ However, a limitation of our study is that we used only the disk diffusion method for the susceptibility test, but not the broth microdilution method or E-test.

Limitation of our study was the lack of confirmative tests with 16S rRNA sequencing for all isolates to affirm the accuracy of the results of MALDI-TOF MS. However, the consistency was noted by randomly selected isolates with *S. mitis* group, which was previously known as the weak point of MALDI-TOF Biotyper system. Besides, our drug susceptibility testing does not use broth microdilution but disc diffusion test. However, this restriction faithfully represented the real-world situation. And the clinical physicians still could receive a lot of assistance of the VGS presentations by knowing their groups identification.

In conclusion, we conducted the largest comprehensive study during the recent years about the different VGS groups and their clinical manifestations, infection sources, concomitant diseases, susceptibility tests, treatments, and outcomes. Different VGS groups had variable underlying diseases, clinical behavior, and drug susceptibility. Therefore, identification of VGS by MALDI-TOF MS has important clinical impacts for physicians.

## Author contributions

**Conceptualization:** Ting-Yi Su, Jang-Jih Lu.

**Data curation:** Ting-Yi Su, Tsui-Ping Liu, Jang-Jih Lu.

**Formal analysis:** Ting-Yi Su, Ching-Tai Huang, Tsui-Ping Liu, Jang-Jih Lu.

**Investigation:** Ting-Yi Su, Ming-Hsun Lee, Ching-Tai Huang, Jang-Jih Lu.

**Methodology:** Ting-Yi Su, Ming-Hsun Lee, Jang-Jih Lu.

**Project administration:** Ting-Yi Su, Jang-Jih Lu.

**Resources:** Ting-Yi Su, Tsui-Ping Liu, Jang-Jih Lu.

**Software:** Ting-Yi Su.

**Supervision:** Ting-Yi Su, Ching-Tai Huang, Jang-Jih Lu.

**Validation:** Ting-Yi Su, Tsui-Ping Liu, Jang-Jih Lu.

**Visualization:** Ting-Yi Su, Jang-Jih Lu.

**Writing – original draft:** Ting-Yi Su.

**Writing – review & editing:** Ting-Yi Su, Ming-Hsun Lee, Jang-Jih Lu.

## Supplementary Material

Supplemental Digital Content
