# Supplementary material for: The clinical impact of patients with bloodstream infection with different groups of Viridans group streptococci by using matrix-assisted laser desorption ionization–time of flight mass spectrometry (MALDI-TOF MS)
Source: Medicine (Baltimore). 2018 Dec 14;97(50):e13607. doi: 10.1097/MD.0000000000013607 (PMC6320099; doi:10.1097/MD.0000000000013607)
Supplement: Supplemental Digital Content [file medi-97-e13607-s001.docx]

Supplement table 1 Comparison of MALDI-TOF Biotyper System identification results of *Streptococcus mitis* group with 16S rRNA sequencing

| Isolates | MALDI-TOF Biotyper system | 16S rRNA sequencing |
| --- | --- | --- |
|  | *Streptococcus oralis* | *Streptococcus oralis* |
|  | *Streptococcus mitis* | *Streptococcus mitis* |
|  | *Streptococcus oralis* | *Streptococcus oralis* |
|  | *Streptococcus mitis* | *Streptococcus mitis* |
|  | *Streptococcus mitis* | *Streptococcus mitis* |
|  | *Streptococcus oralis* | *Streptococcus oralis* |
|  | *Streptococcus oralis* | *Streptococcus oralis* |
|  | *Streptococcus oralis* | *Streptococcus oralis* |
|  | *Streptococcus mitis* | *Streptococcus mitis* |
|  | *Streptococcus oralis* | *Streptococcus oralis* |
|  | *Streptococcus oralis* | *Streptococcus oralis* |
|  | *Streptococcus oralis* | *Streptococcus oralis* |
|  | *Streptococcus oralis* | *Streptococcus oralis* |
|  | *Streptococcus mitis* | *Streptococcus mitis* |
|  | *Streptococcus oralis* | *Streptococcus oralis* |
|  | *Streptococcus oralis* | *Streptococcus oralis* |
|  | *Streptococcus oralis* | *Streptococcus oralis* |
